# Predictive value of ^18^F-FDG PET/CT for evaluating the response to hypofractionated radiotherapy combined with PD-1 blockade in non-small cell lung cancer

**DOI:** 10.3389/fimmu.2023.1034416

**Published:** 2023-02-13

**Authors:** Shan Tang, Yan Zhang, Yunfei Li, Yan Zhang, Yuke Xu, Haoyuan Ding, Yue Chen, Peirong Ren, Hua Ye, Shaozhi Fu, Sheng Lin

**Affiliations:** ^1^ Department of Oncology, The Affiliated Hospital of Southwest Medical University, Luzhou, China; ^2^ Department of Oncology, The First People’s Hospital of Guangyuan, Guangyuan, China; ^3^ Department of Oncology, The People’s Hospital of Luzhou, Luzhou, China; ^4^ Department of Nuclear Medicine, The Affiliated Hospital of Southwest Medical University, Luzhou, China; ^5^ Department of Radiology, The Affiliated Hospital of Southwest Medical University, Luzhou, China

**Keywords:** ^18^F-FDG PET/CT, NSCLC, PD-1 blockade, hypofractionated radiotherapy, predictive value

## Abstract

**Purpose:**

This retrospective study aimed to investigate ^18^F-fluorodeoxyglucose (^18^F-FDG)-positron emission tomography/computed tomography (PET/CT) as a predictor of response to hypofractionated radiotherapy (HFRT) combined with programmed cell death-1 (PD-1) blockade for lung cancer.

**Methods:**

We included 41 patients with advanced non-small cell lung cancer (NSCLC) in this study. PET/CT was performed before (SCAN-0) and one month (SCAN-1), three months (SCAN-2), and six months (SCAN-3) after treatment. Using the European Organization for Research and Treatment of Cancer 1999 criteria and PET response criteria in solid tumors, treatment responses were classified as complete metabolic response (CMR), partial metabolic response (PMR), stable metabolic disease (SMD), or progressive metabolic disease (PMD). Patients were further categorized as those with metabolic benefits (MB; SMD, PMR, and CMR) and those without MBs (NO-MB; PMD). We analyzed the prognosis and overall survival (OS) of patients with new visceral/bone lesions during treatment. Based on the findings, we generated a nomogram to predict survival. Receiver operating characteristics and calibration curves were used to evaluate the accuracy of the prediction model.

**Results:**

The mean OS based on SCANs 1, 2, and 3 was significantly higher in patients with MB and those without new visceral/bone lesions. The prediction nomogram for survival had a high area under the curve and a high predictive value based on the receiver operating characteristics and calibration curves.

**Conclusion:**

^18^FDG-PET/CT has the potential to predict the outcomes of HFRT combined with PD-1 blockade in NSCLC. Therefore, we recommend using a nomogram to predict patient survival.

## 1 Introduction

The treatment of lung cancer is constantly updated, but about 50% of patients have distant metastases at the time of diagnosis. Only 20–30% of patients have the opportunity to undergo surgery, and the overall survival (OS) rate remains low (1, 2). Immune checkpoint inhibitors (ICIs; immunotherapy) targeting programmed cell death-1 (PD-1) have been recently approved for locally advanced and metastatic non-small cell lung cancer (NSCLC) (3), irrespective of the histologic subtype. They have gradually attracted attention and are given in combination with chemotherapy because of their excellent systemic control effect. However, the low patient response to the use of a single drug is a drawback. Some pioneering clinical trials have shown that PD-1 blockade can reactivate the immune system, and encouraging data have been obtained regarding its ability to treat NSCLC (4–8). Many methods have been explored to enhance the systemic efficacy of ICIs [8–10], and a large area of active research is investigating the combination of ICIs with radiation therapy (RT), termed immunoradiotherapy (iRT) (9–11). However, its side effects and increased cost make careful monitoring during therapy necessary. The early recognition of response to therapy or progressive disease could potentially guide treatment alterations, which could benefit the patients.

As a surrogate for intracellular glucose metabolism, ^18^F-fluorodeoxyglucose (^18^F-FDG) positron emission tomography (PET) is used to detect metabolic changes before anatomical changes occur. Previous studies have used PET/CT as an important method for radiotherapy effect evaluation and prognosis judgment of lung cancer (12). The European Organization for Research and Treatment of Cancer (EORTC) 1999 criteria based on standardized uptake value (SUV) (13), as well as the first criterion used to monitor immunotherapy (14), are commonly used to assess the efficacy of treatments based on PET/computed tomography (CT). The PET response criteria in solid tumors (PERCIST V1.0) (15), published in 2009, overcomes the shortcomings of EORTC and uses the liver ^18^F-FDG uptake rate for reference calculation, including the peak standard uptake value-lean (SULpeak) and total lesion glycolysis (TLG).

In recent years, many studies have demonstrated the efficacy of PET/CT in evaluating chemotherapy, RT, immunotherapy, and targeted therapy for NSCLC (16–19). However, the response to iRT is still being explored, and there are few studies on the application of ^18^F-FDG PET/CT in hypofractionated radiotherapy (HFRT) combined with PD-1 blockade in patients with lung cancer. Hence, this study aimed to evaluate the potential of PET/CT for monitoring response to HFRT combined with ICIs in patients with NSCLC and to seek an accurate method for assessing and predicting response.

## 2 Materials and methods

The subjects were patients with pathologically confirmed NSCLC. The inclusion criteria were (1) Eastern Cooperative Oncology Group performance status score ≤ 2; (2) age 18–70 years; (3) previous treatment with at least one line of therapy; (4) at least three measurable lesions on imaging; and (5) serum creatinine level ≤ 2 of the upper normal limit (UNL), aspartate transaminase and alanine transaminase ≤ 3 of the UNL, and hemoglobin level at the lower normal limit. Patients with severe cardiopulmonary dysfunction, active pulmonary tuberculosis, and noninfectious pneumonitis requiring long-term glucocorticoid use and active autoimmune disease were excluded. Forty-one NSCLC patients (31 men and 10 women) admitted to our department between September 2017 and December 2020 who met the inclusion criteria were included in this retrospective study. The decision to administer PD-1 blockade therapy was based on the patient’s financial status. The medication included Nivolumab, Camrelizumab, Sintilimab, Tislelizumab,and Pembrolizuma. The dosage was determined as per the instructions, and the dosage and medication frequency remained unchanged throughout the treatment. The first dose was administered 3–7 days after RT, and the second was given two weeks after the first dose. The PD-1 blockade maintenance dose was administered for one month in the first year, two months in years 2–3, and three months in years 4–5. The RT regimen in this study included stereotactic body radiation therapy (SBRT) (40–50 Gy/5F) and hypofractionated brachytherapy (30 Gy/1F).

### 2.1 ^18^F-FDG PET/CT data acquisition

All 41 patients underwent ^18^F-FDG PET/CT before the start of RT (SCAN-0) and one month after RT (SCAN-1). Of the 41 patients, 26 underwent a third PET/CT scan three months after RT (SCAN-2), and 21 underwent a fourth PET/CT scan six months after RT (SCAN-3).

PET/CT was performed according to the European Association of Nuclear Medicine guidelines version 1.0 (20). Whole-body PET/CT (Philips Gemini TF/16; Philips, Cleveland, OH, USA) was performed after the intravenous administration of ^18^F-FDG (5.55 MBq/kg). The patients fasted for at least six hours before ^18^F-FDG administration, and their blood glucose level was ≤ 11 mmol/L. Low-dose helical CT transmission scanning (pitch, 0.813; current, 100 mA; peak voltage, 120 kV; slice thickness, 5.0 mm) was performed with attenuation correction and lesion localization. PET was performed at 1.5 min per bed position using 19–21 bed positions. ^18^F-FDG PET/CT was performed from the vertex of the head to the feet. Patients held their breath during the chest scans to reduce the impact of respiratory motion on image acquisition and ensure the accuracy of the results.

### 2.2 ^18^F-FDG PET/CT data analysis

PET/CT images were analyzed by two nuclear medicine physicians using a workstation. Target lesions were selected according to the PERCIST criteria. A maximum of two lesions were selected in each organ when multiple measurable lesions were available, and no more than five target lesions with highest SUV were selected. PET-based target lesion delineation was carried out with an SUV of 2.5 as the initial threshold. The maximum SUV (SUVmax), metabolic tumor volume (MTV), SULpeak, and TLG were calculated for the target lesions.

### 2.3 Response evaluation

The patient responses to RT were evaluated by ^18^F-FDG PET/CT as per the EORTC (13) and PERCIST criteria. Both criteria classify tumor responses as progressive metabolic disease (PMD), stable metabolic disease (SMD), partial metabolic response (PMR), or complete metabolic response (CMR). Stable disease represents a satisfactory outcome following immunotherapy since, in contrast to conventional chemotherapy, it can be durable, and survival rates related to stable disease are comparable to those associated with response. Based on the responses, patients were further divided into two groups: those demonstrating metabolic benefit (MB; including SMD, PMR, and CMR) and those demonstrating NO-MB (NO-MB; including patients with PMD).

In addition, we focused on new visceral/bone lesions in three scans after treatment and analyzed the prognosis of these patients separately.

### 2.4 Consistency evaluation of short-term response

The Kappa test was used to assess the consistency of PET scan evaluation results in each period, and the total Kappa value ranged from 0 to 1. When the Kappa value was less than 0.4, it indicated that the consistency was poor, and when the Kappa value was between 0.4 and 0.75, it indicated that the consistency was moderate. When the Kappa value is greater than or equal to 0.75, the consistency between the two is high. A separate analysis was conducted for those cases with a difference in evaluation results.

### 2.5 Depth of response

Depth of response (DpR) was defined as the percentage change in SUVmax of the target lesion from baseline. Following SCAN-1, patients were divided into three groups based on the percentage change in SUVmax in the tumor target lesions: group 1 (G1; < 30% decrease), group 2 (G2; 30–50% decrease), and group 3 (G3; > 50% decrease). G1 included patients with no change in SUVmax.

### 2.6 Construction of prediction model

Imaging parameters were collected one month before and one month after treatment. From SCAN-0, we collected the sum of PET parameters of target lesions, named SUVmax0, SULpeak0, TLG0 and MTV0. The imaging parameters of the same lesion were recorded again in SCAN-1 as SUVmax1, SULpeak1, TLG1, and MTV1. The changes in the imaging parameters were calculated as △SUVmax = SUVmax0 - SUVmax1, △SULpeak = SULpeak0 - SULpeak1, △TLG0 = TLG0 - TLG1, and △MTV = MTV0 - MTV1. The average baseline PET parameters were also calculated and recorded as the baseline SUVmax, baseline SULpeak, baseline TLG, and baseline MTV. Patient age, sex, pathological type, Eastern Cooperative Oncology Group (ECOG) status, and response evaluation (MB/NO-MB) were all included in the preliminary screening characteristics. In the preliminary preparation work, we confirmed that the immunotherapy and RT regimens of the patients had no significant correlation with OS, so they were not included in the preliminary features for screening.

### 2.7 Statistical analysis

OS was recorded and defined as the time from RT to death from any cause. Survival curves according to each variable were estimated using Kaplan-Meier and log-rank tests. The Cox proportional hazards regression model was used for the univariate and multivariate analyses of preliminary characteristics. The multivariate model used the AIC criterion to screen the variables, and the results were visualized using a nomogram. The receiver operating characteristic curve was used to analyze the value of the lipopograph model to determine the prognosis. Internal consistency was verified using bootstrap and demonstrated using the calibration curve. Statistical analyses were performed using R, version 3.5.0. software. Statistical significance was set at p < 0.05.

## 3 Results

### 3.1 Patient characteristics

**Table 1** summarizes the characteristics of patients included in this analysis. The mean age of the 41 patients was 57.7 ± 9.3 years (range 37–75 years). All patients had stage III–IV NSCLC; 34.1% (14/41) had squamous cell carcinoma and 65.9% (27/41) had adenocarcinoma. While 65.85% (27/41) of the patients were treated with nivolumab, 19.51% (8/41) received camrelizumab, 7.32% (3/41) received sintilimab, 4.88% (2/41) received tislelizumab, and 2.44% (1/41) received pembrolizumab. While 29.27% (12/41) of patients were treated with hypofractionated brachytherapy, 70.73% (29/41) of them were treated with SBRT.

**Table 1:** Patient’s clinical characteristics at baseline.

	N	Percent(%)
Total patients	41	100.00
Age in years, mean ± SD	57.7 ± 9.3	
Sex		
Male	31	75.61
Female	10	24.39
Histology		
Squamous cell carcinoma	14	34.15
Adenocarcinoma	27	65.85
PD-1 blockades		
Nivolumab	27	65.85
Camrelizumab	8	19.51
Sintilimab	3	7.32
Tislelizumab	2	4.88
Pembrolizumab.	1	2.44
Radiotherapy		
HFBT	12	29.27
SBRT	29	70.73

HFRT, hypofractionated brachytherapy; SBRT, stereotactic body radiation therapy.

### 3.2 Response evaluation

#### 3.2.1 SCAN-1

**Figure 1:**
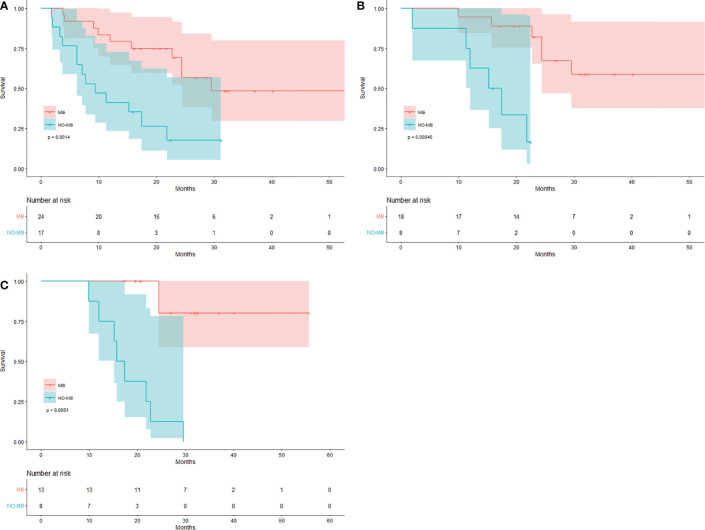
Kaplan-Meier curves for response evaluation. Patients with MB and NO-MB on **(A)** SCAN-1, **(B)** SCAN-2, and **(C)** SCAN-3.

SCAN-1 findings were evaluated for all 41 patients. According to the EORTC criteria, 24 patients showed MB (0 CMR, 18 PMR, and 6 SMD), whereas 17 patients had NO-MB (PMD). Using the PERCIST criteria, 24 patients had MB (3 CMR, 13 PMR, and 8 SMD), whereas 17 had NO-MB (PMD). Based on the clinical follow-up data on SCAN-1, the median OS of patients with PMD was 9.4 months (mean 13.3 months), with 13 (76.47%) deaths by the end date. In patients with MB, the median OS was 29.5 months (mean 36.0 months), with 10 (41.67%) deaths. The difference between the group MB and group NO-MB was statistically significant (log-rank p = 0.0014). The Kaplan-Meier plots for OS are shown in **Figure 1A**.

#### 3.2.2 SCAN-2

For the 26 patients who underwent three PET/CT examinations, the SCAN-2 results showed that while 18 patients had MB (3 CMR, 11 PMR, and 4 SMD) according to the EORTC criteria, 8 had NO-MB (PMD). According to the PERCIST criteria, 18 patients had MB (6 CMR, 7 PMR, and 5 SMD) and 8 had NO-MB (PMD). Based on the clinical follow-up data on SCAN-2, the mean OS of patients with PMD was 15.4 months with 6 (75%) deaths, while in those with MB, it was 41.8 months with 6 (33.33%) deaths. The difference between the group MB and group NO-MB was statistically significant (log-rank p = 0.00046). The Kaplan-Meier plots for OS are shown in **Figure 1B**. The median OS was unavailable because the mortality rate of patients with MB did not exceed 50%. These data are summarized in **Table 2**.

**Table 2:** Treatment response of the patients investigated in the study.

Patient no.	Treatment response evaluation			MB/NO-MB			Whether there are new visceral/bone lesion(s)(Yes/No)			OS(months)	Status
	SCAN-1	SCAN-2	SCAN-3	SCAN-1	SCAN-2	SCAN-3	SCAN-1	SCAN-2	SCAN-3		
1	PMR	PMR	PMD	MB	MB	NO-MB	NO	NO	YES	9.9	dead
2	PMR	PMR	PMR	MB	MB	MB	NO	NO	NO	31.8	alive
3	SMD	SMD	SMD	MB	MB	MB	NO	NO	NO	24.4	dead
4	SMD	PMD	PMD	MB	NO-MB	NO-MB	NO	NO	YES	12.0	dead
5	PMD	PMD	PMD	NO-MB	NO-MB	NO-MB	NO	NO	NO	17.4	dead
6	PMR	PMR	PMR	MB	MB	MB	NO	NO	NO	32.1	alive
7	PMD	PMD	PMD	NO-MB	NO-MB	NO-MB	NO	YES	YES	21.8	dead
8	PMR	CMR	CMR	MB	MB	MB	NO	NO	NO	55.6	alive
9	PMR	CMR	CMR	MB	MB	MB	NO	NO	NO	37.0	alive
10	PMR	PMR	PMR	MB	MB	MB	NO	NO	NO	40.1	alive
11	PMD	PMD	PMD	NO-MB	NO-MB	NO-MB	NO	YES	YES	15.2	dead
12	PMR	SMD	PMR	MB	MB	MB	NO	NO	NO	20.6	alive
13	SMD	SMD	SMD	MB	MB	MB	NO	NO	NO	19.6	alive
14	PMR	PMR	PMD	MB	MB	NO-MB	NO	NO	YES	15.7	dead
15	PMD	PMR	PMR	NO-MB	MB	MB	NO	NO	NO	31.1	alive
16	PMR	PMR	PMD	MB	MB	NO-MB	NO	NO	NO	22.7	dead
17	SMD	PMR	PMR	MB	MB	MB	NO	NO	NO	24.4	dead
18	SMD	CMR	CMR	MB	MB	MB	NO	NO	NO	32.5	alive
19	PMR	PMR	PMD	MB	MB	NO-MB	NO	NO	NO	29.5	dead
20	SMD	SMD	SMD	MB	MB	MB	NO	NO	NO	17.3	alive
21	PMR	PMR	PMR	MB	MB	MB	NO	NO	NO	26.9	alive
22	PMD	PMD		NO-MB	NO-MB		YES	YES		11.3	dead
23	PMD	PMD		NO-MB	NO-MB		YES	YES		22.4	alive
24	PMD	PMD		NO-MB	NO-MB		NO	NO		3.0	dead
25	PMD	PMD		NO-MB	NO-MB		NO	NO		15.9	alive
26	PMR	PMR		MB	MB		NO	NO		23.0	alive
27	PMD			NO-MB			YES			6.2	dead
28	PMD			NO-MB			YES			3.3	dead
29	PMR			MB			NO			4.0	dead
30	PMD			NO-MB			YES			3.7	dead
31	PMR			MB			NO			3.8	dead
32	PMD			NO-MB			YES			1.8	dead
33	PMR			MB			NO			9.1	dead
34	PMD			NO-MB			YES			7.1	dead
35	PMR			MB			NO			21.7	alive
36	PMD			NO-MB			NO			9.4	dead
37	PMD			NO-MB			NO			6.2	dead
38	PMD			NO-MB			YES			7.7	dead
39	PMD			NO-MB			YES			15.9	alive
40	PMR			MB			NO			28.6	alive
41	PMR			MB			NO			16.1	alive

PMD, progressive metabolic disease; SMD, stable metabolic disease; PMR, partial metabolic response. MB, metabolic benefit, including SMD, PMR, and CMR. NO-MB; no metabolic benefit, including patients with PMD.

#### 3.2.3 SCAN-3

SCAN-3 revealed that 13 patients had MB (3 CMR, 7 PMR, and 3 SMD) and 8 patients had NO-MB (PMD) based on the EORTC criteria. According to the PERCIST criteria, 13 patients had MB (6 CMR, 3 PMR, and 4 SMD) and 8 had NO-MB (PMD). Based on the clinical follow-up data on SCAN-3, the mean OS of patients with PMD was 18.0 months with 8 (100%) deaths, and in those with MB, it was 49.4 months with 2 (15.38%) deaths. The difference between the group MB and group NO-MB was statistically significant (log-rank p < 0.0001). The Kaplan-Meier plots of OS are shown in **Figure 1C**. The median OS could not be obtained because the mortality rate of the patients with MB did not exceed 50%.

#### 3.2.4 New visceral/Bone lesion(s)

**Figure 2:**
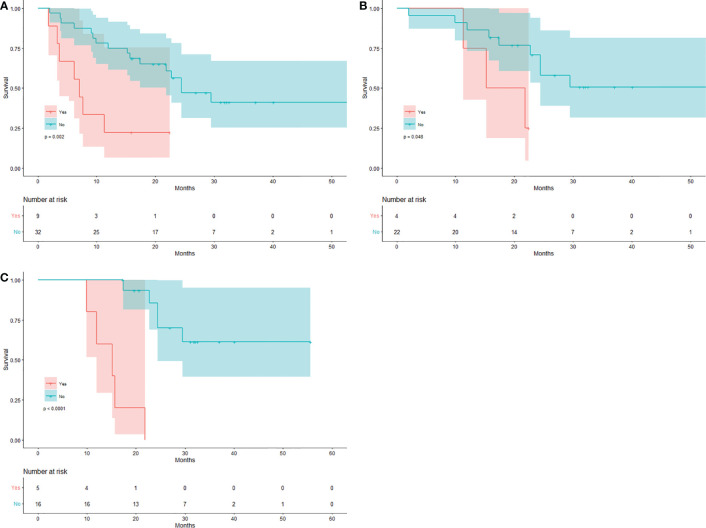
Kaplan-Meier curves for the new visceral/bone lesion(s). Patients with/without new visceral/bone lesion(s) on **(A)** SCAN-1, **(B)** SCAN-2, and **(C)** SCAN-3.

The median OS in 9 patients with new visceral/bone lesions on SCAN-1 was 7.1 months (mean 9.5 months), with 7 (77.78%) deaths by the end date of follow-up. The median OS in the other 32 patients without new lesions was 24.4 months (mean 32.2 months), with 16 (50%) deaths. The difference between the two groups was statistically significant (log-rank p = 0.002). The Kaplan-Meier plots for OS are shown in **Figure 2A**. The mean OS in 4 patients with new visceral/bone lesions on SCAN-2 was 17.7 months, with 3 (75%) deaths. The mean OS in the other 22 patients without new lesions was 37.5 months, with 9 (40.91%) deaths. The difference between the two groups was statistically significant (log-rank p = 0.048). The Kaplan-Meier plots of OS are shown in **Figure 2B**. The mean OS in 5 patients with new visceral/bone lesions on SCAN-3 was 14.9 months with 5 (100%) deaths, while in the other 16 patients without new lesions, it was 43.4 months with 5 (31.25%) deaths. The difference between the two groups was statistically significant (log-rank p < 0.0001). The Kaplan-Meier plots for OS are shown in **Figure 2C**. For SCAN-2 and SCAN-3, the median OS could not be obtained because the mortality rates in patients with MB did not exceed 50%.

### 3.3 Consistent evaluation of short-term response

#### 3.3.1 Consistency comparison of SCAN-1 and SCAN-2

**Table 3** presents Consistency of short-term response of PET/CT. A total of 26 patients were included in the evaluation, including 18 patients with MB and 8 with NO-MB as assessed by SCAN-1, as well as 18 patients with MB and 8 with NO-MB as assessed by SCAN-2. However, 1 patient was evaluated as having PMD (NO-MB) in SCAN-1 due to several new lesions in the lung. In SCAN-2, all the new lesions disappeared, and the tumor burden of the primary lesion was reduced. The patient’s OS was 31.1 months, and he was still alive by the end follow-up date, with good clinical benefit. Therefore, his disease status was considered as pseudoprogression and evaluated as PMR (MB) in SCAN-2. One patient diagnosed with SMD (MB) in SCAN-1 was diagnosed with PMD (NO-MB) in SCAN-2. This patient exhibited lymph node metastasis on a PET scan 3 months after treatment, and the SUVmax value of the original lesion increased. The response evaluation results of two PET/CT treatments were consistent (p = 0.000029, Kappa value was 0.819). Therefore, PET evaluation at 1 month after treatment can roughly predict efficacy at 3 months after treatment.

**Table 3:** Consistency of short-term response of PET/CT.

	KAPPA value	P
SCAN-1 and SCAN-2	0.819	0.000029
SCAN-1 and SCAN-3	0.33	0.091
SCAN-2 and SCAN-3	0.553	0.005

The consistency of the response evaluation results of SCAN-1 and SCAN-2 were generally consistent. The consistency of the response evaluation results of SCAN-1 and SCAN-3 was low. The consistency of the response evaluation results of SCAN-2 and SCAN-3 was moderate.

#### 3.3.2 Consistency comparison of SCAN-1 and SCAN-3

A total of 21 patients were included in the evaluation, including 17 patients with MB and 4 patients with NO-MB on SCAN-1, as well as 13 patients with MB and 8 patients with NO-MB on SCAN-3. Five patients with MB (4 PMR, 1 SMD) at SCAN-1 had progression at SCAN-3, including 3 new visceral/bone metastases. All 3 patients died, with an average OS of 12.5 months. The other 2 patients with only new lymph node metastases had an average OS of 26.1 months. One patient who was evaluated as having PMD (NO-MB) in SCAN-1 was evaluated as having PMR (MB) in SCAN-2 and SCAN-3, which was the same patient with the above pseudoprogression. The consistency of the response evaluation results of two PET/CT treatments was low (p = 0.091, Kappa value was 0.33).

#### 3.3.3 Consistency comparison of SCAN-2 and SCAN-3

A total of 21 patients were included in the evaluation, including 17 with MB and 4 with NO-MB on SCAN-2 and 13 with MB and 8 with NO-MB on SCAN-3. Four patients with MB (4 PMR) on SCAN-2 had new metastases on SCAN-3. The mean OS times of 2 patients with new bone metastases and 2 patients with new lymph node metastases were 12.8 months and 26.1 months, respectively. The consistency of the response evaluation results of two PET/CT treatments was moderate (p = 0.005, Kappa value was 0.553).

#### 3.3.4 Depth of response

**Figure 3:**
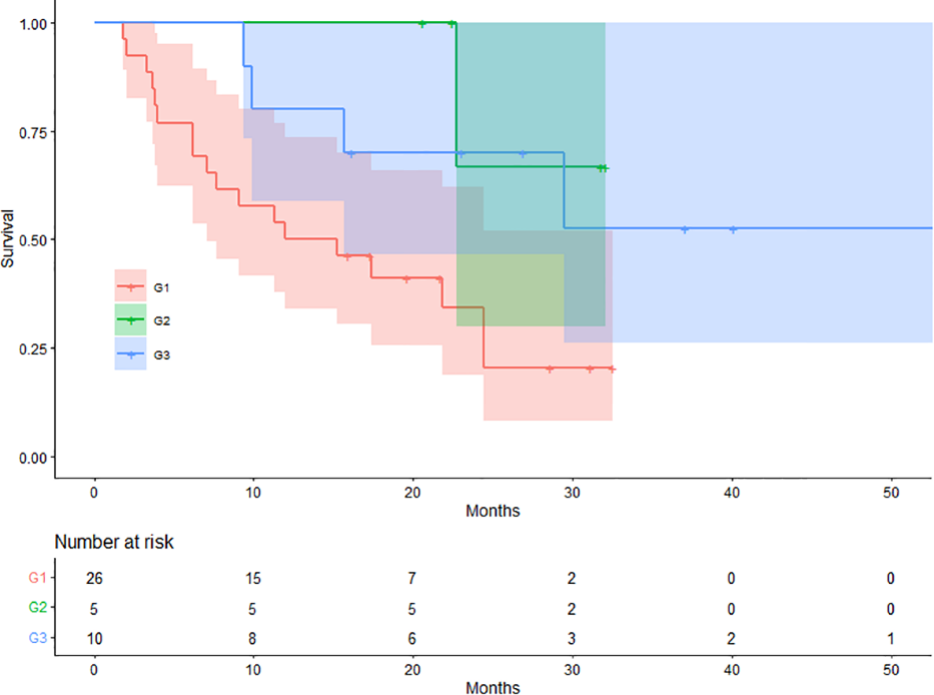
Kaplan-Meier curves for the depth of response.

On SCAN-1, 26 (63.41%), 5 (12.20%), and 10 (24.39%) patients were classified into G1, G2, and G3, respectively. The mean OS values for patients in the G1, G2, and G3 groups were 15.98, 28.97, and 37.85 months, respectively. There were 18 (69.23%), 1 (20.00%), and 4 (40.00%) deaths in the G1, G2, and G3 groups, respectively. The difference in OS between the three groups was statistically significant (log-rank p = 0.021). The Kaplan-Meier plots for OS are shown in **Figure 3**.

#### 3.3.5 Prediction model

**Figure 4:**
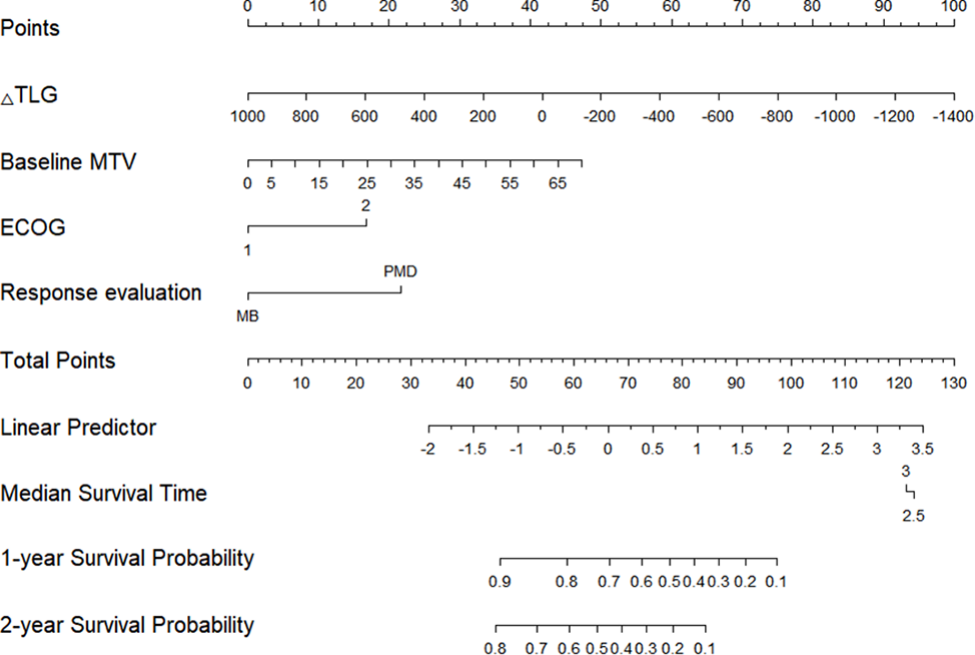
The nomogram based on the combination of clinical and PET/CT features.

**Figure 5:**
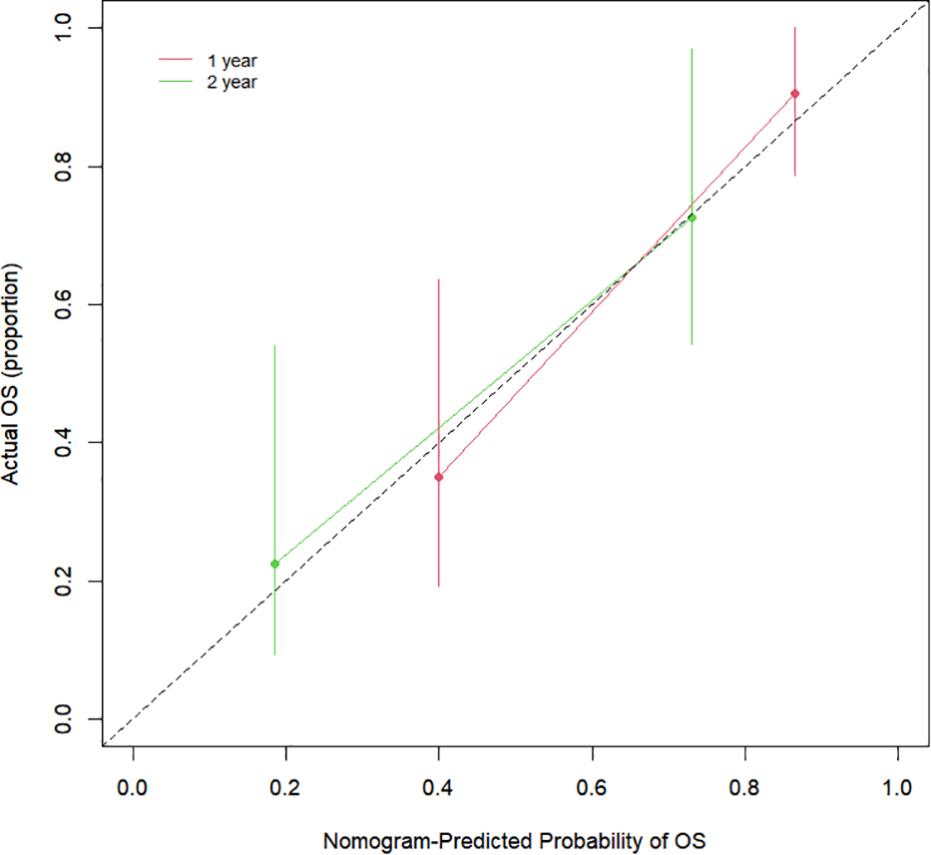
Calibration curves for the clinical parameters combined with the PET/CT features model.

**Figure 6:**
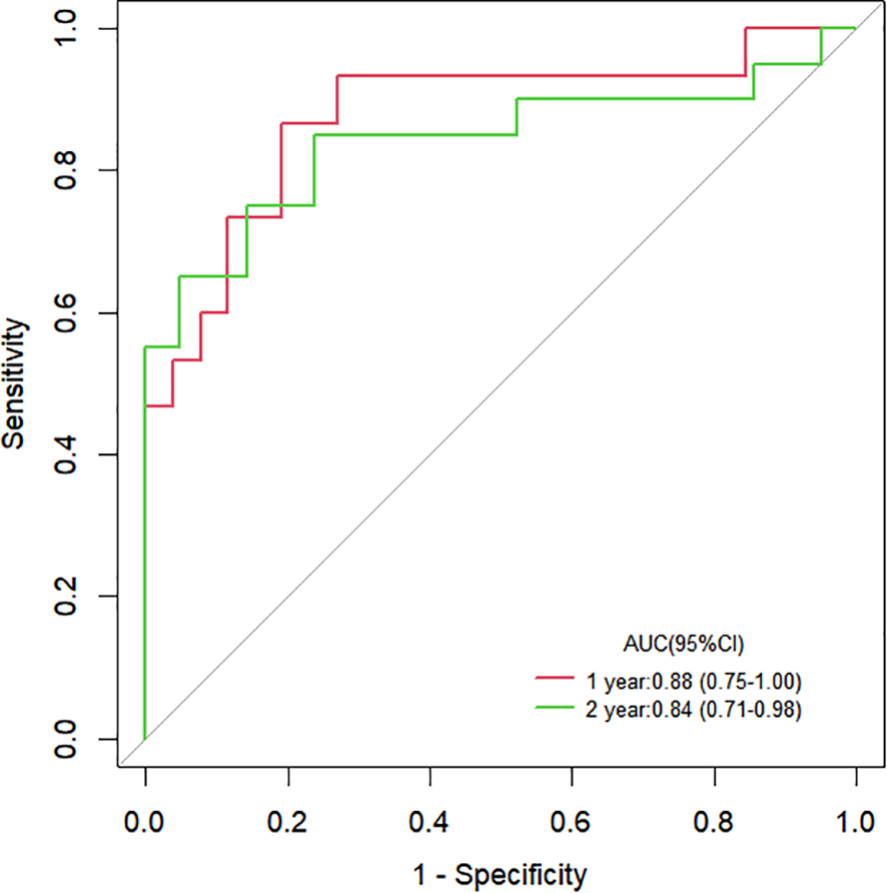
ROC curves for the clinical parameters combined with the PET/CT features model.

The Cox proportional hazards regression model results showed that ΔTLG, baseline MTV, ECOG performance status, and response evaluation were independent factors for death after correction for other factors (p < 0.05) (**Table 4**). The risk of death was 0.997 (0.996–0.999) times higher for each unit increase in ΔTLG, 1.042 (1.009–1.075) times higher for each unit increase in baseline MTV, and 2.762 (1.028–7.421) times higher for patients with an ECOG of 2 than those with an ECOG of 1. The risk of death was 3.703 (1.441–9.516) times greater in patients with PMD than in those with MB. These four features were used to establish a nomogram model (**Figure 4**). The C index of the model was 0.801 (95% confidence interval [CI]: 0.731–0.884). **Figures 5**, **6** show the internal validation of the model. Based on the receiver operating characteristics curve analysis, the model had a high area under the curve (1 year: 0.88, 95% CI: 0.75–1.00, 2 years: 0.84, 95% CI: 0.71–0.98) (**Figure 5**). The calibration curves for the 1-year and 2-year OS prediction models were closer to the 45° diagonal, indicating that the model predicted survival rates closer to the actual rates (**Figure 6**).

**Table 4:** The results of COX proportional hazards regression model.

Characteristic	Univariate analysis		Multivariate analysis		
	*P*	*HR(95%CI)*	*P*	*HR(95%CI)*	
Baseline SUVmax	0.809	0.986(0.876-1.109)			
△SUVmax	0.004	0.948(0.914-0.983)		
Baseline SULpeak	0.770	0.974(0.817-1.161)		
△SULpeak	0.010	0.942(0.901-0.986)		
Baseline TLG	0.029	1.005(1-1.009)		
△TLG	0.046	0.999(0.997-1)	0.001	0.997(0.996-0.999)
Baseline MTV	0.044	1.025(1.001-1.051)	0.011	1.042(1.009-1.075)
△MTV	0.248	0.993(0.981-1.005)		
Gender male v.s female	0.820	0.898(0.353-2.282)		
Age	0.765	0.993(0.946-1.042)		
Pathological pattern squamous carcina v.s adenocarcinoma	0.375	0.668(0.274-1.629)		
ECOG 2 v.s 1	0.008	3.478(1.386-8.73)	0.044	2.762(1.028-7.421)
Response evaluation PMD v.s MB	0.003	3.773(1.589-8.958)	0.007	3.703(1.441-9.516)

Baseline SUVmax, mean SUVmax of target lesion in SCAN-0; △SUVmax, SUVmax0 - SUVmax1; Baseline SULpeak, mean SULpeak of target lesion in SCAN-0; △SULpeak, SULpeak0 - SULpeak1; Baseline TLG, mean TLG of target lesion in SCAN-0; △TLG0, TLG0 - TLG1; Baseline MTV, mean MTV of target lesion in SCAN-0; △MTV, MTV0 - MTV1.

## 4 Discussion

In recent years, some clinical trials have shown that iRT could potentially be utilized for any stage of NSCLC. For metastatic cases, there is randomized evidence to support the addition of RT to immunotherapy (21–23). For locally advanced non-metastatic cases, the randomized PACIFIC trial demonstrated the efficacy of combining definitive RT with subsequent immunotherapy (24). Lastly, for early-stage NSCLC, there are several randomized trials aiming to evaluate stereotactic RT with or without adjuvant immunotherapy (e.g.NCT03110978, NCT03446547, NCT03833154, NCT03924869, NCT04214262). Some investigators suggest that SBRT is more effective in activating the body’s anti-tumor immunity than conventional fractionated RT and is the best “companion” for combined ICIs (25). A phase I study evaluated multisite SBRT followed by pembrolizumab for metastatic solid tumors, including NSCLC, and the results showed that the combination therapy had a high control rate in both post-RT and non-RT metastatic tumors. The RECIST-based overall response rate was 13.2%. Moreover, the median OS and progression-free survival were 9.6 months (95% CI: 6.5 months–undetermined) and 3.1 months (95% CI: 2.9–3.4 months), respectively (26). In our preliminary clinical trial study, we enrolled 31 patients with advanced lung cancer pathologically confirmed to have progressive disease, and 23 patients who completed the treatment were evaluated. At the 1-year follow-up, no patients had developed grade ≥ 3 pneumonitis. The overall objective response and complete remission rates were 39.13% and 13.04%, respectively. The 1-year OS and median progression-free survival were 60.9% and 6 months, respectively (27).

To our knowledge, the published data, though limited, indicate that the combination treatment has considerable promise in future NSCLC treatment. There is therefore an urgent need for reliable monitoring programs and predictors. Although PD-L1 positivity is enriched in populations with clinical benefits, PD-L1 testing alone appears to be insufficient for patient selection (28). Since there are no validated biomarkers that help identify NSCLC patients who are more likely to benefit from the combination of ICIs and RT, therapeutic decisions currently rely on imaging combined with a clinical evaluation. In 1999, for the first time, the EORTC defined PET criteria for standard response assessment using ^18^F-FDG PET for solid tumors (13). Ten years later, the refined PERCIST system was developed based on additional literature (15). In addition, due to a limited cohort of patients, new immune-related PET criteria have been proposed, mainly focusing on melanoma or lymphoma (29–32). However, the response pattern for RT combined with immunotherapy by imaging is still poorly understood. The immune-related PET criteria for immunotherapy were established based on small-sized clinical studies, and the accuracy of evaluation still needs to be confirmed. Therefore, based on the clinical trials conducted by our group, the EORTC and PERCIST criteria were used for response evaluation. We established a preliminary training set to determine the response prediction value of PET/CT for HFRT combined with PD-1 blockade.

Christos et al. (14) used the EORTC criteria for the first time to evaluate the response to immunotherapy and found that the response to early PET (after two cycles of treatment) could effectively predict the outcomes of the intermediate PET (four cycles). They could determine a predictive value for the prognosis of PMD and SMD by studying 22 patients with melanoma treated with epirimizumab who underwent PET/CT before and two and four cycles after treatment. In this study, it was also found that the evaluation results of SCAN-1 and SCAN-2 were generally consistent and the response of PET at 1 month after treatment could roughly predict the response at 3 months after treatment. Therefore, we suggest that patients undergo only one PET scan within 3 months after treatment, this therapeutic strategy should reduce both economic burden and radiation side effects for patients. Meanwhile, both criteria were highly predictive of OS in studies of NSCLC patients treated with RT plus chemotherapy, with a high agreement in efficacy evaluation between the two (Kappa value = 0.95) (33). A previous study on small cell lung cancer treated with RT and chemotherapy reached similar conclusions, with both criteria in complete agreement, and a significant difference was seen in OS between the CMR and No CMR groups (p = 0.0431) (34). However, in this study, due to the addition of PD-1 blockade, both SMD and PMR patients received good clinical benefits; therefore, we grouped the patients into PMD and No PMD groups. When patients were classified into the CMR, PMR, SMD, and PMD groups, we found slight differences in the evaluation based on the two criteria. When patients were further dichotomized into the MB (No PMD) and NO-MB (PMD) groups, the findings were identical with both criteria. PET was able to classify most patients in all periods, although some were still misclassified.

In addition, we explored other indicators associated with OS to identify prognostic factors. In this study, the number of new visceral/bone lesions was low and was seen in 9/41(21.95%), 4/26(15.38%), and 5/21(23.81%) patients in the SCAN-1, SCAN-2, and SCAN-3 groups, respectively. Although the lesions could not be counted effectively, a preliminary trend could be seen in the Kaplan-Meier survival curve. Compared to patients with no metastases, those with metastases had significantly longer OS times. Previous studies have suggested that the presence of new visceral/bone lesions was the strongest surrogate indicator of poor prognosis following treatment with ICIs in patients with NSCLC; only 5/20 patients achieved durable clinical benefits. Sensitivity, specificity, positive predictive value, negative predictive value, and Youden’s indices for predicting no benefit were 71.4%, 82.8%, 75%, 80%, and 0.54, respectively. However, there was still a high number of responding patients who were misclassified (positive predictive value = 75%) (18). Therefore, we hypothesized that new visceral/bone lesions might indicate a poor prognosis in NSCLC patients receiving RT combined with ICIs. These patients should be removed from the group and either switched to regimens that combine other treatments or enrolled in other clinical trials.

We aimed to identify patients with a poor prognosis as early as possible, and therefore, we focused on the correlation between PET parameters in SCAN-1 and prognosis. This study classified patients using SUVmax decline rates of 30% and 50% as the cutoff points. An SUVmax decline rate of > 50% was associated with significantly longer OS, suggesting that the DpR is correlated with prognosis in early PET after treatment to some extent. However, the potential of △SUV (before and after treatment) as a prognostic indicator remains unclear. In a study on nasopharyngeal carcinoma patients (35), Qi et al. used percent SUV decline during RT to assess radiosensitivity. They reported that a 70% SUVmax decline after two cycles of treatment was a good cutoff point for PET to predict tumor regression after RT and chemotherapy in these patients. In a study of 46 patients with advanced lung adenocarcinoma who received gefitinib-targeted therapy, they found that compared to patients with △SUV% ≥ 25% (PMD), the survival time was significantly prolonged in those with △SUV% < -25% (including CMR and PMR) (10.6/18.4, p = 0.000) but not in patients with -25% ≤ △SUV% < 25% (SMD) (10.6/10.7, p = 0.088) (36).

In summary, although the indicators explored in this study were correlated with prognosis to a certain extent, some patients were still misclassified. Establishing a personalized prediction model is necessary to ensure precise treatment and accurate prediction of which patients would benefit from the treatment. Based on the data from this clinical trial, we established a training set to help develop subsequent clinical trials. PET/CT provides information on metabolic parameters, such as SUV, SUL, MTV, and TLG. Lin et al. (37) demonstrated that > 50% decreases in lymph node SUVmean, MTV, and TLG during RT are prognostic predictors of locally advanced head and neck squamous carcinoma. Changes in MTV and TLG before and after treatment are accurate and independent prognostic indicators in various tumors, including nasopharyngeal, esophageal, pancreatic, and ovarian cancers (38–41). In a retrospective study of NSCLC patients receiving chemotherapy, Moon et al. found that patients with a ΔTLG of > 50% had a longer progression-free survival after one cycle of chemotherapy (42). In another prospective study of 37 patients with NSCLC treated with RT and chemotherapy, Huang et al. (43) found that patients with more significant changes in SUVmax and MTV before and after treatment had better treatment sensitivity. In this study, ΔTLG, baseline MTV, ECOG performance status, and response evaluation were screened using the Cox proportional hazards regression model to generate a nomogram graph and a model to predict survival with an ideal predictive value for patients treated with HFRT combined with PD-1 blockade.

## 5 Limitations and conclusion

This study has some limitations. First, like the previous studies, it was a small-sized, single-center clinical study. Currently, available response evaluation criteria are based on single treatment modalities. Studies with larger sample sizes are urgently needed to evaluate RT combined with immunotherapy as a prospective treatment option and to establish precise and complete evaluation criteria. Second, the prediction model in this study used only the metabolic parameters of PET and not PET radiomics to analyze the texture-structure parameters of the images. PET-based radiomics is also a critical prognostic tool for patients with NSCLC after RT or immunotherapy.

In conclusion, our preliminary data show that PET/CT can correctly classify most patients using the EORTC and PERCIST criteria, and it is recommended that only one PET scan be performed 3 months after treatment. Additionally, new visceral/bone lesions and DpR may be prognostic indicators, and a predictive model of survival probability with an optimal predictive value has been established. To the best of our knowledge, this is the first trial to investigate the potential of PET/CT for monitoring and predicting the outcomes of HFRT plus PD-1 blockade therapy in patients with NSCLC. A PET/CT scan after treatment could be a reliable indicator of patient outcomes and should be investigated further.

We are conducting a multicenter prospective study of HFRT combined with PD-1 blockade for NSCLC, including more patients, to explore further the value of PET/CT for evaluating the response to RT combined with ICIs. In the future, we plan to improve the validation set of the prediction model and develop a complete and accurate patient monitoring system.

## Data availability statement

The original contributions presented in the study are included in the article/**Supplementary Material**. Further inquiries can be directed to the corresponding author.

## Ethics statement

The studies involving human participants were reviewed and approved by the Affiliated Hospital of Southwest Medical University Clinical Trial Ethics Committee. The patients/participants provided their written informed consent to participate in this study. Written informed consent was obtained from the individual(s) for the publication of any potentially identifiable images or data included in this article.

## Author contributions

Conception and design: SL, and ST. Administrative support: SL. Provision of study materials or patients: YZ (2nd author), YL and YZ (4th author). Collection and assembly of data: YX, HD, and YC. Data analysis and interpretation: ST, PR, HY, and SF. Manuscript writing: ST. Final approval of manuscript: SL. All authors contributed to the article and approved the submitted version.
